# Barriers and facilitators for referring women with positive perinatal depression screening results in China: a qualitative study

**DOI:** 10.1186/s12884-023-05532-6

**Published:** 2023-04-05

**Authors:** Wenqing Xue, K K Cheng, Lu Liu, Qiao Li, Xin Jin, Jingmin Yi, Wenjie Gong

**Affiliations:** 1 Xiangya School of Public Health, Central South University, 110 Xiangya Road, 410078 Changsha, Hunan China; 2 Institute of Applied Health Research, University of Birmingham, B15 2TT Birmingham, UK; 3 Department of Psychiatry, University of Rochester, 14642 Rochester, USA

**Keywords:** Perinatal depression, Referral, Barriers, Facilitators

## Abstract

**Background:**

Timely screening and referral can improve the outcomes of perinatal depression (PND). However, uptake rates of referral after PND screening are low in China and the reasons are unclear. The aim of this article is to explore the barriers and facilitators for referring women with positive results of PND screening in the Chinese primary maternal health care system.

**Methods:**

Qualitative data were collected from four primary health centers located in four different provinces of China. Each of the four investigators conducted 30 days of participant observations in the primary health centers from May to August 2020. Data were collected via participant observations and semi-structured in-depth interviews with new mothers who had positive results of PND screening, their family members, and primary health providers. Two investigators analyzed qualitative data independently. A thematic analysis was conducted, and data were framed using the social ecological model.

**Results:**

A total of 870 hours of observation and 46 interviews were carried out. Five themes were identified: individual (new mothers’ knowledge of PND, perceived need to seek help), interpersonal (new mothers’ attitudes towards providers, family support), institutional (providers’ perception of PND, lack of training, time constraints), community (accessibility to mental health services, practical factors), and public policy (policy requirements, stigma).

**Conclusions:**

The likelihood of new mothers accepting PND referral is related to factors in five areas. Intervention strategies can be developed around these themes and may include educating new mothers and their families about PND, training primary health providers to improve their awareness of condition and indication for referral, building mental health support in routine postpartum home visits, and providing support through mobile technology.

**Supplementary Information:**

The online version contains supplementary material available at 10.1186/s12884-023-05532-6.

## Background

In 2020, the National Health Commission of China (NHC) announced a work plan calling for routine screening for perinatal depression (PND) and encouraging medical institutions to provide follow-up professional support [1]. PND is common during pregnancy or postnatal period and affects approximately 11.9% of mothers worldwide. The prevalence is higher in low- and middle-income countries compared to high-income countries [2]. In mainland China, the prevalence is estimated to be 16.3% [3]. As PND has serious impact in mothers and offspring [4–6], early identification and timely support may be helpful. Therefore, many countries and professional bodies have recommended screening for PND.

For screening to work, however, identification of women with positive screening results needs to be followed by timely referral and effective interventions. One outstanding and unexpected feature about referral after positive screens is the low uptake rates. Uptake rates here means the proportion of women who accept referral and try to access mental health services among those who are offered referrals. A previous systematic review including all eligible articles in English published before 2015 found that only an average of 22% of women with positive PND screening results had at least one mental health visit when referred to care [7]. Our team updated this review in 2019 and found a pooled uptake rate of 43% [8]. We found three studies conducted in China. Possibly due to the differences in the nature of interventions and the location of research, the three studies showed a wide range of uptake rates, ranging from 0.4 to 76% [9–11]. Suboptimal uptake has clear negative impact on the effectiveness of screening.

There are studies that explore the factors influencing referral for women with positive PND screening, including the facilitators and barriers experienced by women [12–21]. However, these studies had several limitations. First, most were conducted in countries that have established routine PND screening and their results may not be applicable elsewhere (e.g. China). Second, most studies used questionnaires or interviews to collect relevant data, but given the sensitivity around psychological disorders, ‘snapshot’ assessments using standardized questionnaires or interviews may be inadequate. Participant observation over a longer period may benefit from a trusting relationship and yield more realistic information [22]. Finally, accepting referral is essentially an act of health service utilization and involves not only new mothers, but also family members and healthcare providers. However few studies have included all of them as study participants [23, 24].

In this study, we engaged multiple participants (new mothers, family members, and primary care providers) and conducted qualitative study (participant observations and interviews) in the primary care setting in China. The aim of this article is to explore the barriers and facilitators for referring women with positive results of PND screening in the Chinese primary maternal health care system.

## Methods

The protocol has been published in ResearchGate before this study: https://www.researchgate.net/publication/341615859_A_single_protocol_for_The_barriers_and_promotion_strategies_for_the_identification_and_referral_of_women_at_high_risk_for_perinatal_depression--a_qualitative_study.

### Settings and participants

Investigators purposely selected four primary health centers in urban (site 1, site 2) and rural (site 3, site 4) areas from four provinces in China as study settings for participant observation and interviews. These four provinces are ranked 1st (site 1), 11th (site 2), 19th (site 3) and 20th (site 4) in the 2020 total GDP ranking of 31 provinces [25]. Participant were purposively-selected and included in in-depth interviews: (1) women at high risk of PND, pregnant or within one year after delivery, aged ≥ 18 years, and within the jurisdiction of the four primary health centers. In this study, women scoring 10 or more on the Chinese version of Edinburgh Postpartum Depression Scale (EPDS) or had a positive response on item 10 (self-injury thoughts) or both were regarded as being at high risk of PND [26]. As there was no routine screening for PND in China, investigators administered the EPDS when new mothers were waiting for maternal and child healthcare. (2) family members who accompanied the interviewed women at the primary care centers, including their husbands/partners, mothers-in-law and mothers. (3) providers who worked in maternal or child health in the four centers, including maternal health providers, child healthcare providers, obstetricians, gynecologists and healthcare managers.

### Data collection

This study received approval from the Medical Ethics Committee of Xiangya School of Public Health, Central South University (No. XYGW-2020-44). Four investigators (Q.L, W.X, L.L, X.J) undertook the participant observation and interviews. They were postgraduate students majoring in maternal and child health and have experience of maternal mental health research. In addition, they have received online training by an experienced sociologist.

Access to the four primary health centers was negotiated with managers and informed consent was obtained in advance of planned participant observation. Across the four study sites, four investigators (Q.L, W.X, L.L, X.J) who worked as junior staff and shadowed the maternal health providers on their day-to-day work, each spent approximately one month to carry out participant observations from May to August 2020. They began with general observation to familiarized themselves with the daily work of maternal health providers and the setting of the centers. Then they focused on gathering experiences in relation to maternal health providers’ daily work. The participant observations were conducted in, but not limited to, health care department for women in primary health centers or new mothers’ home. Daily notes were written up on participant observations.

As the investigators had opportunities to approach every eligible participant, they purposely selected and explained the background, content, benefits and risks of our study to eligible interviewees. After obtaining their verbal consent, the time and place (either a private room in primary health centers or participants’ homes) of interviews were agreed with the participants. Before interviews, written formal consent was obtained and demographic data were collected from all interviewees. During personal in-depth interviews, the investigators asked a series of open-ended questions based on the interview guides (see Additional file 1) which was developed after literature review. The outline of interviews involved perceptions and attitudes toward PND, experiences of offering/receiving PND referrals, barriers and facilitators to offering/receiving PND referrals. Interviews were audio-recorded using professional recording equipment. Interviewees received 7.8 USD as a gift of thanks via WeChat transfer immediately after interviews. Investigators transcribed the interviews verbatim from the audio recordings within 24 h after interview and sent to interviewees for accuracy check within 3 days after interviews.

### Data analyses

Qualitative data were analyzed using thematic analysis described by Braun and Clarke [27]. The social ecological model was applied to examine influencing factors for referral [28]. First, all the transcripts and observation notes were read at least three times, allowing the researchers to familiarize themselves with the data. After this, initial codes were generated by segmenting data and coding it verbatim. Then we analyzed the results of initial coding and aggregated them into themes. The themes were then reviewed and refined in their correspondence to the codes that composed them. We named each theme based on the most clearly captured aspects of the data. Finally, a report was written to show the study results. In data analysis, the social ecological model was used to frame themes and show how each theme related to the others. This model is widely used in public health to influence health outcomes and effect behavior change at the individual, interpersonal, institutional, community, and public policy levels, while promoting referral is itself a change in medical-care-seeking behavior [28]. To conduct theoretical saturation test, two of the authors reviewed the data independently. One (W.X) analyzed approximately two-thirds of the data first and the remaining one-third of the data was analyzed by another author (J.Y). Both authors believed that theoretical saturation had been achieved. All data collected were anonymized. The qualitative data were analyzed using NVivo 12.0.

This article is reported in line with the Standards for Reporting Qualitative Research Checklist (see Additional file 2).

## Results

Across four sites, 870 hours of observations were carried out (site 1: 240 hours; site 2: 225 hours; site 3: 195 hours; site 4:210 hours) and 183,046 words of observational notes were produced. A total of 46 participants were included in the interviews, including 26 new mothers, 5 family members, and 15 primary health care providers. The demographic characteristics of interviewees are shown in Tables 1, 2 and 3. Interviews lasted between 16 and 176 min (average of 49 min). The interviews produced a total of 523,341 words of textual information.

**Table 1:** Demographic data of new mothers (n = 26)

Characteristic	N
Role	
Pregnant women	10
Postpartum women	16
Ethnicity	
Han	24
Ethnic minority	2
Location	
Urban area	18
Rural area	8
Education	
Middle school or lower	9
High school	4
College	13
Job	
Unemployed	15
In work	11
Household income (yuan/per month)	
1-2000	4
2001–5000	7
5001–10,000	9
10,001–20,000	6
Medical insurance	
Yes	22
No	4
Living situation	
Nuclear family	6
Nuclear family with parents in law	14
Nuclear family with parents	4
Living alone or living with children	2
Depression history	
No	26
Whether had heard of perinatal depression/postnatal depression	
Yes	21
No	5
Age ($\bar x \pm s$)	28.46 ± 5.46
Score of EPDS ($\bar x \pm s$)	13.08 ± 3.30

**Table 2:** Demographic data of family members (n = 5)

Characteristic	N
Role	
Husband	3
Mother-in-law	1
Mother	1
Ethnicity	
Han	5
Location	
Urban area	4
Rural area	1
Education	
Middle school or lower	3
High school	1
College	1
Job	
In work	5
Household income (yuan/per month)	
2001–5000	2
5001–10,000	2
10,001–20,000	1
Whether had heard of perinatal depression/postnatal depression	
Yes	4
No	1

**Table 3:** Demographic data of primary health providers (n = 15)

Characteristic	N
Gender	
Men	2
Women	13
Location	
Urban area	8
Rural area	7
Position	
Maternal health provider	5
Child health provider	3
Obstetrician or gynecologist	3
Manager	4
Years of work	
< 3 years	4
4-10 years	2
> 10 years	9
Seniority	
Junior	5
Intermediate	8
Senior	2

During observation, primary healthcare providers did not seem to pay much attention to new mothers’ mental health. None of the interviewed providers had any experiences of referral. Only two providers noted that they would advise new mothers to go to hospital if they found women at high risk of PND and most of the providers said they would just talk with new mothers to comfort them.

“(During the whole home visit,) neither Yu (maternal health provider) nor Sheng (maternal health provider) asked about the mother’s recent emotional state, and there were no relevant words in the whole conversation.” [observation notes #02].

“Today, when the new mother scored high on the scale during the visit, L (maternal health provider) would chat with the new mother, suggesting that they talk to family members if things are not going well.” [observation notes #01].

None of the new mothers sought or received mental health services before the in-depth interviews. After being informed of positive screening results, none indicated a wish to see a mental health specialist. New mothers relied more on informal help, such as self-regulation or seeking help from friends and family. A few women used non-specialist resources, such as seeking help from primary health providers or through online information.

“Just talking with my colleagues and then they enlightened me.” [new mother #11].

We identified several barriers and facilitators of PND referral at individual, interpersonal, institutional, community and public policy levels.

### Individual level

#### New mothers’ knowledge of PND

Knowledge of PND was the first individual level factor. Although the majority of interviewed women reported that they had heard of perinatal depression or postnatal depression, their knowledge was often associated with serious adverse outcomes they heard in the media and did not have a deep understanding of PND itself. Many women believed that depression would not happen to themselves or attributed their feelings to natural changes during perinatal period rather than depression, which to some extent reduced women’s tendency to seek help.

“I don’t have much idea of what depression is.” [new mother #07].

“I think most of it is caused by the environment and family, not by the psychological (problems) of new mothers.” [new mother #01].

Similar to new mothers, most family members also reported that they did not know what PND was or what measures may be available. They were not able to distinguish between normal and abnormal emotions, especially when women did not actively reveal their emotions. Besides, three family members believed that new mothers never experienced depressed mood or any depression could be alleviated through self-regulation.

“I don’t know, I… You, what to do? If you do not tell me and do not show me, what can I do, right? I can’t help it.” [family member #03].

Among the few interviewed women who had good understanding of PND, concerns about the negative impact of PND on their normal lives prompted them to receive appropriate mental health services.

“I am afraid after I gave birth to my second child I will do something wrong, since I did have suicidal thoughts during my first pregnancy.” [new mother #09].

#### Perceived need to seek help

New mothers’ perceived need to seek help is a facilitating factor as recognition and awareness of difficulties and symptoms can help triggering help seeking behavior. Self-recognition or being told by family members and providers about mood abnormalities was important.

“Once after a fight with my in-laws, the symptoms got worse and worse, and (I thought I) might have a problem and then I came here for help.” [new mother #02].

### Interpersonal level

#### New mothers’ attitudes towards providers

Some women expressed dissatisfaction with providers’ attitude and perceived providers as focusing on physical problems and not mental health. They were seen as often being in a hurry, making them unwilling to sharing their feelings. Besides, several interviewed women expressed mistrust towards providers’ ability to address problems and preferred to relieve depression through self-regulation or family support.

“Many doctors just take care of what they should do for you and don’t tell you anything beyond physical examination.” [new mother #07].

“I don’t think it is a good idea to seek help from doctors. The only thing that might be useful is, as I said before, if you are really in bad mood, just talk to a friend, it might be much better.” [new mother #27].

However, some women demonstrated trust in their perinatal health care providers or mental health providers to help alleviate or resolve their emotional problems.

“Then I may go to the doctor, because (friends and relatives) cannot help me simply by talking with me. So I can only go to the doctor, who can help me solve the problem.” [new mother #08].

#### Family support

Family support had impact on women’s help seeking behavior. Some interviewed new mothers reported lack of understanding and support from their families regarding mental health problems. Some were even prevented from seeking mental health services. Three family members expressed their opinion of the results of PND screening, and their reactions reflected their reluctance to believe in it. However, two interviewed family members supported PND referral because they were concerned about the negative impact of depression.

“My mother also can’t understand. She think because my parents-in-laws are helping me with my baby, I don’t have…I don’t have the pressure of looking after the child. My work is also quite good and secure (in a state-owned enterprise). She said ‘why would you have postpartum depression?’ ” [new mother #06].

“Depression definitely has severe impact on the mother, and if it affects the baby, that is pretty bad.” [family member #03].

### Institutional level

#### Providers’ perception of PND

Recognition of the importance of PND and the positive significance of referral is a key motivation for primary care providers to carry out related screening and referral. Over half of the interviewed providers expressed such views.

“She (maternal health providers) said it is quite a good thing to take care of new mothers’ psychological condition, in case the woman is really depressed, our timely feedback may save her life, and it would be a really good thing to have done.” [observational notes #02].

#### Lack of relevant training

Interviewed providers said they had little training regarding PND and had no knowledge of the process of screening and referral. More than half of them expressed a thought that there was no need for referral since depression could be self-limiting or alleviated through family support which was in line with family members’ opinions.

“I don’t think it’s needed. It is not all that serious; it may last a short period of time and then it will ease up on its own.” [provider #13].

#### Time constraints

Perceived time constraints hindered the offer of referral. Primary health care providers have heavy workload, especially maternal health workers. Some providers thought they were too busy to listen.

“Because patient volume is already large, we can’t finish those work (physical examinations), not to mention the extra work (mental health care).” [provider #06].

“I think their (maternal health providers) work is really complicated. LY (maternal health provider) has to do not only maternal health care work, but also gynecology outpatient services and menopausal health care work.” [observation notes #01].

### Community level

#### Accessibility to mental health services

Limited accessibility to mental health services was an important impediment to help seeking. Difficulties in accessing health care facility may reduce women’s use of services. Long distance from home to health care providers and long waiting time were perceived as unhelpful.

“There are no mental health clinics and doctors around here, even if someone wanted to use services, they could not.” [provider #14].

#### Practical factors

The fear of high costs was one of the practical barriers to seeking help, especially for women who were unemployed and had no personal income. In addition, lack of time and difficulties in finding a caretaker for their baby were also practical barriers to seeking mental health services.

“Now everything costs money, go (hospital) all at once (will spend a lot of money), 100 yuan is nothing now.” [new mother #19].

“It’s not very convenient (to go to the doctor), (we) have to take care of the children.” [new mother #14].

### Public policy level

#### Policy requirements

At the time of our study, the NHC did not require primary health centers to conduct PND screening and referral. The lack of clear policy requirements weakened the initiative of primary health providers. Most interviewed providers were reluctant to take on the task when there was no requirement about it.

“Generally speaking, we do not care about things which are not part of our work. For example, we are required to do postnatal visits, so we will do it.” [provider #06].

#### Stigma

Stigma also seemed to impact mental health services use. Prevailing societal norms emphasize the positive side of delivery and motherhood, expecting mothers to be happy and strong. These norms may stigmatize mothers with PND and reduce the possibility of seeking help. The negative impact of stigma may be particularly serious in Chinese cultural context, which emphasize “Don’t wash your dirty linen in public”.

“I feel that if I talk to doctors about this topic, they will tell me I am a bit pretentious.” [new mother #10].

“I would feel ashamed, or say, or say I don’t want people to know I’m mentally ill, I’m a mental patient.” [new mother #17].

Although the majority of new mothers reported being affected by stigma when confronted with psychological problems, six women reported that depression was more common for women in pregnancy and childbirth and did not consider seeking help for depression as something that was stigmatized or unknowable to outsiders.

“I am not …I am not afraid that others know that I have psychological problems.” [new mother #07].

## Discussion

In this study, we found that primary healthcare providers and new mothers with positive screening results offered or received few referrals for perinatal depression respectively. Only two of the fifteen interviewed primary healthcare providers indicated that they would advise women to seek professional help. None of the 26 interviewed new mothers showed any intention of seeking mental health services after being screened for perinatal depression. The referral proportion in this study was far below the rates reported in previous studies in other countries [7, 8]. Ours was a small sample but a similar finding was reported in our previous study [9]. This may be due to the fact that China does not have a well-developed mechanism for identifying and referring for PND.

Most new mothers, their family members and primary health providers thought abnormal emotions could be alleviated through “self-regulation” or family support. This may be related to traditional Chinese culture, as well as the importance of support among family members [29]. It is suggested that referral is not the only solution and future studies could make use of the ability of self-regulation and family support as the directions of PND interventions [30, 31].

Some women were reluctant to seek help because they were dissatisfied with providers’ attitude. This suggests the importance of doctor-patient communication. Studies showed that women value the relationship with providers and that the establishment of a harmonious relationship with providers is key to women feeling “being cared for”, especially for women during perinatal period [32]. Therefore, training of providers should ensure that they would encourage women to talk about their feelings and would listen carefully.

A previous study explored the role of informal help-seeking behaviors such as seeking family support [33]. They found that support from family members was one of the main sources of social and emotional support received by women and had a positive effect on women’s emotional relief and treatment seeking. Some mothers in this study did not feel supported by their families regarding their emotional problems. This may be related to a lack of understanding of PND among family members. Education about PND could reduce this barrier, such as encouraging families to attend antenatal classes.

Although primary health providers undertake the bulk of maternal health care works, most of those interviewed in this study lack mental health related knowledge. Training is necessary to equip them with basic skills to recognize and to refer for PND [7]. The lack of knowledge and training may be related to the fact that routine screening had not yet been introduced in China at the time of this study. We expect this barrier to be alleviated when relevant health policies are enacted. However, before the formal national rolling out of screening, an easy-to-implement referral tool may be helpful. To increase feasibility, the identification and referral of PND needs to be integrated into the routine duties of primary care workers, which currently include postnatal home visits. We are currently developing such a tool.

Training and supervising non-specialists to provide mental health care may improve access to mental health services [31]. Such task-sharing is advocated by the World Health Organization and meta-analyses have shown its effectiveness in common perinatal mental health disorders, particular in low-and middle-income countries [34, 35]. In addition, mobile health (mHealth) can also improve the accessibility of mental health resources [36, 37]. mHealth has increased in popularity since the onset of the COVID-19 pandemic mHealth and could overcome barriers such as the lack of local relevant resources and the need to travel long distances to access medical care.

Difficulties in finding a caretaker for baby hindered use of mental health services and home-visiting may be one solution [38]. Primary maternal healthcare providers in China are required to provide twice home-visits within seven days and 30 days after delivery respectively [39]. Though the first month after delivery is a high-risk period of PND [40], the tradition of “doing-the-month” makes Chinese women inaccessible during this period [41, 42], as the women are expected to adhere to behavioral practice including the restriction of activity, especially going out. Home-visiting provides a valuable opportunity for the identification and referral PND women.

Stigma is a common barrier to seeking professional help for depression in several studies [43–45]. This may be related to the limited knowledge of PND among new mothers. For example, the most mentioned symptoms - ‘low mood’ and ‘suicidal thoughts or behavior’ - are among the most prevalent and extreme symptoms, while the more common symptoms such as poor quality of sleep, was less mentioned. On the other hand, some women attributed emotional abnormalities to external pressures such as family conflicts but, ignored its physiological basis, which might have also contributed to the stigma [46]. Such culturally related factors need to be alleviated primarily through long-term, general health education and promotion for patients and the public. In addition, the integration of mental health services into routine perinatal healthcare to avoid highlight the ‘independence’ of mental health services may reduce stigma [47].

There are several limitations to this study. First, though we approached every eligible participant during study, not all of them accepted the invitations for our interview. We also did not collect demographic characteristics of those who refused and the reasons for refusal. However, qualitative study emphasizes informative in terms of samples, and its results require that information reached saturation. Therefore, we believe that our study still provides the in-depth explanations and meanings related to the barriers and facilitators for referring women with positive results of PND screening that would be helpful in addressing the problem of suboptimal referral. Second, some literature suggested that education level is associated with service use for PND. However, over half of the interviewed new mothers in this study had a college or bachelor’s degree, which may limit the extrapolation of our findings. Finally, this study lacked observations and interviews with women who received referrals after screening. Future studies should include women who were successfully referred to mental health services after screening to explore the experiences and influencing factors during the referral process.

## Conclusion

This study identified several barriers and facilitators at individual, interpersonal, institutional, community, and public policy levels that can affect women’s behavior of seeking and accepting mental health services during perinatal period. There may be benefits in undertaking educational programs which target new mothers and their families. Providers should receive adequate training and try to provide mental health services during home-visiting or through mobile health.

## Electronic supplementary material

Below is the link to the electronic supplementary material.

**Additional file 1.** Interview Guides

**Additional file 2.** Standards for Reporting Qualitative Research, SRQR

## Data Availability

The datasets supporting the conclusions of this article are not publicly available due to reasons of privacy and confidentiality, however, deidentified data may be available from the corresponding author on reasonable request.

## Abbreviations

NHC: The National Health Commission of China
PND: Perinatal depression
EPDS: Edinburgh Postpartum Depression Scale
