# Supplementary material for: Barriers and facilitators for referring women with positive perinatal depression screening results in China: a qualitative study
Source: BMC Pregnancy Childbirth. 2023 Apr 5;23:230. doi: 10.1186/s12884-023-05532-6 (PMC10074342; doi:10.1186/s12884-023-05532-6)
Supplement: Supplementary file 1 — Additional file 1. Interview Guides [file 12884_2023_5532_MOESM1_ESM.docx]

**Additional file 1. Interview Guides**

**To new mothers**

1. Can you tell us what you think is perinatal depression？

2. Did you experience any change in mood during pregnancy or after giving birth? You can talk to me about anything that made you happy or unhappy.

3. Did any providers pay attention to your mental health during your pregnancy or after you giving birth? Was there any help offered?

4. When you were in a bad mood, how did you deal with it? And why?

5. If a provider offered you a referral to mental health services, would you be willing to? Why?

6. What kind of help would you like to receive? Why?

**To family members**

1. Can you tell us what you think is perinatal depression？

2. Did your wife/daughter/daughter-in-law experience any change in mood during pregnancy or after giving birth?

3. Did she talk to you about it? How do you respond?

4. When she were in a bad mood, how did you deal with it? And why?

1. If a provider offered she a referral to mental health services, what would you say? Why?

**To primary healthcare providers**

1. Can you tell us what you think is perinatal depression？

2. What measures have been taken to manage new mother’ mental health in this center?

3. Have you ever met with a new mother who might has perinatal depression in work? Can you describe it in detail?

4. How do you identify if a new mother has perinatal depression? Why?

5. How do you deal with it when you find a new mother who might has perinatal depression? Why?
